# Activation of the GLP-1 Receptor Signalling Pathway: A Relevant Strategy to Repair a Deficient Beta-Cell Mass

**DOI:** 10.1155/2011/376509

**Published:** 2011-05-22

**Authors:** Bernard Portha, Cécile Tourrel-Cuzin, Jamileh Movassat

**Affiliations:** Laboratoire B2PE (Biologie et Pathologie du Pancréas Endocrine), Unité BFA (Biologie Fonctionnelle et Adaptive), Equipe 1, Université Paris-Diderot et CNRS EAC 4413, Bâtiment BUFFON, 5ème étage, pièce 552A, 4, Rue Lagroua Weill Hallé, Case 7126, 75205 Paris Cedex 13, France

## Abstract

Recent preclinical studies in rodent models of diabetes suggest that exogenous GLP-1R agonists and DPP-4 inhibitors have the ability to increase islet mass and preserve beta-cell function, by immediate reactivation of beta-cell glucose competence, as well as enhanced beta-cell proliferation and neogenesis and promotion of beta-cell survival. These effects have tremendous implication in the treatment of T2D because they directly address one of the basic defects in T2D, that is, beta-cell failure. In human diabetes, however, evidence that the GLP-1-based drugs alter the course of beta-cell function remains to be found. Several questions surrounding the risks and benefits of GLP-1-based therapy for the diabetic beta-cell mass are discussed in this review and require further investigation.

## 1. The Rationale for Restoration of Beta-Cell Mass in Diabetic Patients

Both type 1 and type 2 diabetes are characterised by deficits in beta-cell mass (~99% deficit in long-standing type 1 diabetes, ~65% deficit in long-standing type 2 diabetes [1]). There is little doubt regarding the importance of increased autoimmune-mediated beta-cell death in type 1 diabetes, and recent studies in type 2 diabetes suggest that the frequency of beta-cell apoptosis is also significantly increased, although other factors cannot be excluded, such as the failure of beta-cell mass to expand adequately in response to rising secretory demands by adapting beta-cell replication and neogenesis. Loss of beta-cells in both types of diabetes implies that restoration of endogenous insulin secretion and normalisation of hyperglycemia in such patients might be accomplished through the supplementation of islet cells. Indeed, hyperglycemia in both types of diabetes is reversed by pancreas transplantation, and intraportal transplantation of isolated islets temporarily restores glucose control. Unfortunately, replacement of beta-cell mass by islet or pancreas transplantation is associated with both surgical morbidity and the adverse effects of chronic immunosuppression. Some of the risks and side effects, including ischemic and enzymatic damage caused by the islet isolation and purification protocol as well as the concerns of thrombosis and portal hypertension induced by transplanting islets into the liver portal vein, are intrinsic to the islet transplantation procedure itself [2]. Moreover, there is an insufficient supply of pancreases available for the increasing number of people with diabetes, thus preventing the widespread implementation of this intervention. There is, therefore, a need for alternative approaches for restoring functional beta-cell mass in patients with diabetes. 

Conceivable approaches to achieve beta-cell supplementation consist of restoring an endogenous source and/or implanting an autologous- or nonautologous-derived source. At present, there are different strategies under investigation: (1) transplantation of beta cells generated in vitro from nonautologous embryonic stem cells, (2) transplantation of beta-cells generated in vitro from patient's own adult stem cells, and (3) stimulation of beta-cell regeneration in vivo from patient's own endogenous cell sources.

 An alternative strategy for the restoration of beta-cell mass in patients with diabetes is to foster in vivo beta-cell regeneration from patient's endogenous cell sources. There is now evidence that beta-cell mass is dynamic and capable of undergoing adaptive changes in response to different secretory demands. In humans, beta-cell mass increases by ~50% in obesity, and both insulin secretion and beta-cell mass have been shown to increase in pregnant women [3]. Likewise, beta-cell mass in rodents increases by ~2.5-fold towards the end of pregnancy and is rapidly decreased through increased apoptosis and reduced replication postpartum. In humans, the overall capacity for beta-cell replication is much lower than in rodents, and very few replicating beta cells (one cell in ~50 islets of ~100 beta-cells each per cross-section) can be found in adult human pancreas [1]. There is, however, a capacity for increased beta-cell replication in humans: beta-cell replication has been reported to be more than ten times higher in human pancreas adjacent to gastrin-producing tumours [4] and in the pancreas of an old patient with recent-onset type 1 diabetes [5]. Indeed, the emerging understanding of beta-cell growth in the adult, either from precursor cells found in the pancreatic ducts or/and from residual beta cells, holds the promise of developing new strategies for stimulating beta-cell regeneration. Such approach necessitates the delivery of appropriate growth factors to these cells to obtain a full beta-cell phenotype. GLP-1 could be one of the most promising candidates for doing so. The following sections review our current understanding of the therapeutic potential of the GLP-1 receptor (GLP-1R) agonists for the diabetic beta-cell population.

## 2. Activation of the GLP-1R Signalling Pathway and Beta-Cell Functions

GLP-1 replenishes beta-cell insulin stores via increased insulin mRNA stability, gene transcription, and biosynthesis. It stabilizes mRNA encoding preproinsulin, thereby stabilizing and upregulating its expression [6, 7]. GLP-1 increases *insulin* gene transcription and biosynthesis via activation of both PKA-dependent and -independent signalling pathways. PDX-1, the most extensively studied insulin transcription factor, is a key effector for the GLP-1R signaling pathway on *insulin* gene transcription and biosynthesis, as well as differentiation, proliferation, and survival of the beta cell. GLP-1 has been shown, both in vitro and in vivo, to be involved in regulation of PDX-1 by increasing its total protein levels, and its translocation to the nucleus, followed by its binding to the A-box element and the GG2 element of the rat and human insulin promoters and resultant increase in activity of the *insulin* gene promoter in beta cells [8–13]. The regulation of PDX-1 by GLP-1 mainly occurrs via cAMP/PKA-dependent signaling pathway [10]. Nevertheless, GLP-1 triggers expression and nuclear localization of PDX-1 involves the phosphorylation of FoxO1 via transactivation of the EGFR and PI-3K/PKB pathway, resulting in deactivation and nuclear exclusion of FoxO1 and consequent disinhibition of Foxa2-dependent pdx-1 gene promoter activity [14, 15]. In addition, FoxO1 and PDX-1 mutually exclude each other from the nucleus of the beta cell [14]. The GLP-1R signaling pathway also mediates *insulin* gene transcription via basic region-leucine zipper transcription factors that are related structurally to the transcription factor CREB, and these directly bind to CRE sites on the insulin gene promoter. This effect is independent of Gs*α*, cAMP/PKA, and PKC and may be mediated by the 90-kDa ribosomal S6 kinase and mitogen- and stress-activated protein kinase family of CREB kinases [16, 17]. 

GLP-1 is one of the most potent substances known to stimulate glucose-induced insulin secretion (GIIS), and its stimulatory activity is exerted via binding to its receptor on beta cells. This binding results in activation of adenylyl cyclase with consequent production of cAMP and subsequent activation of PKA and the Epac family. GLP-1-mediated activation of PKA results in phosphorylation of the SUR1 KATP channel subunit via an ADP-dependent mechanism, facilitating its closure [18]. This is followed by membrane depolarization and triggering of the insulin secretory pathway. Treatment with the PKA inhibitor 8-bromoadenosine-3′,5′-cyclic mono-phosphorothioate, Rp-isomer [19], or H89 [18] abolishes GLP-1-induced inhibition of the KATP channels. SUR1(−/−) islets lack an insulin secretory response but exhibit a normal rise in cAMP to GLP-1, implicating cAMP-dependent PKA-independent signal transduction pathway [20, 21]. It is now clear that the action of cAMP produced by GLP-1 signaling is mediated not just by PKA, but also by Epac2 [22, 23], and Epac2 also inhibits the function of KATP channels in rodent and human beta-cells via interaction with SUR1 [24, 25]. A recent study has also demonstrated that the scaffold protein, *β*-arrestin-1, facilitates GLP-1-stimulated cAMP production via interaction with GLP-1R [26]. GLP-1 signaling also antagonizes voltage-dependent K^+^ (Kv) channels via cAMP/PKA-dependent pathway in beta cells, which prevents beta-cell repolarization by reducing Kv currents [27]. However, MacDonald et al., [27] identified a role of PI-3K with subsequent activation of PKC*ζ* in the antagonism of the Kv current by GLP-1. This occurred via epidermal growth factor receptor (EGFR) transactivation, not via the G protein-regulated isoform p110*γ* [27]. L-type Ca^2+^ channels are also phosphorylated by PKA, leading to increase of their open probability and enhancement of Ca^2+^ influx [28–30]. Activation of GLP-1R also increases intracellular Ca^2+^ through Ca^2+^-release from the endoplasmic reticulum via the inositol 1,4,5 triphosphate receptors activated by PKA and the ryanodine receptors activated by Epac2 [31, 32]. A recent study has indicated that GLP-1 elevates intracellular Ca^2+^ concentration via stimulation of the nicotinic acid adenine dinucleotide phosphate and cyclic ADP-ribose production [33], catalyzed by cyclic ADP-ribose cyclases, that stimulates glucose-induced Ca^2+^ mobilization [34]. Direct effects of GLP-1 signaling on insulin-containing vesicles have also been described. Lester et al. [35] proposed a mechanism by which changes in insulin secretion are associated with phosphorylation of the vesicle-associated protein synapsin-1 by PKA followed by dephosphorylation by calcineurin [35]. PKA may also regulate the vesicle priming through the phosphorylation of RIM proteins [36]. It is known that RIM proteins bind Rab3a, which serves to tether the vesicle to the plasma membrane [37] and also bind Munc13-1 to create a link between synaptic vesicle tethering and priming [38]. RIM proteins also bind Epac2, and this binding participates in the regulation of docking and fusion of insulin-containing vesicles to the plasma membrane [39, 40]. In addition, Epac2 interacts with Picollo, a RIM2-interacting protein on insulin-containing vesicles, in a Ca^2+^-dependent manner [41]. 

Intracellular cAMP levels have long been recognized to be critical for normal GIIS (glucose-competence) [42]. Thus, receptors linked to cAMP production, such as the GLP-1R play an important role to control cellular cAMP levels. It has been very recently proposed that the biological process regulated by GLP-1 to control the beta-cell glucose competence was dependent on the level of IGF-1R expression and on IGF-2 secretion [43]. Finally, since GLP-1 also stimulates the expression of GLUT2 transporters and glucokinase, which determine the rate of glycolysis, it helps to confer glucose competence to beta cells and thereby increase the efficacy (maximal effect) and potency (threshold concentration) of glucose as a stimulus for insulin secretion [44]. However, this interpretation is questionable in the light of recent extensive data indicating that GLP-1 barely affects beta-cell intermediary metabolism and that metabolic signalling does not significantly contribute to GLP-1 potentiation of GIIS [45].

## 3. Pharmacological Activation of the GLP-1R Signalling Pathway in Glucose-Insensitive Diabetic Beta Cells

Enhancement of GIIS from the beta cell is one of principal goals for treatment of patients with T2D. Because the mechanism underlying the insulinotropic action of GLP-1 in an in vitro model of glucose-unresponsive beta cells (dispersed rat beta cells) has been shown to involve activation of adenylate cyclase and cAMP production [42], we investigated the effect of GLP-1 stimulation on cAMP production and GIIS in GK/Par rats with spontaneous T2D [46]. Diabetic GK/Par islets were able to amplify their cAMP content in response to GLP-1 in the presence of high glucose, and this was associated with a strong insulin release with restitution of their insulin secretory competence to glucose [47]. GLP-1-stimulated cAMP generation was instrumental in the GLP-1-triggered insulin release at high glucose since insulin release became no longer reactive to GLP-1 when AC isoforms were acutely blocked by the AC blocker dd-Ado. Since we also demonstrated that GK/Par beta cells suffer from some degree of cAMP-resistance, one may conclude that GLP-1 at pharmacological dosage, is able to generate within the GK/Par beta cell, cAMP levels high enough to cope for the reduced effectiveness of cAMP [47]. Furthermore, we report that GLP-1 also normalizes GIIS in islets from n-STZ rats (another recognized model of rat diabetes with glucose-unresponsive beta cells) [48]. This suggests that restoration by GLP-1 of glucose responsiveness in the diabetic beta cell is not restricted to the GK model, but is probably a more generalized mechanism.

## 4. Activation of the GLP-1R Signalling Pathway and Beta-Cell Growth/Survival

Repair or expansion of the beta-cell population can be achieved through stimulation of beta-cell proliferation and/or neogenesis, and slowing the rate of beta-cell apoptosis. Abundant in vitro and in vivo studies have shown that GLP-1, and its analogs such as Exendin-4 (Ex-4) are capable of inducing beta-cell proliferation in normal rodent islets and insulinoma cell lines [49–52]. This proliferative seems to involve the activation of immediate early genes such as c-jun, junD, nur77, and c-fos [53], and the implication of different intermediary signaling molecules such as PI3K, PKB/Akt, and PKCzeta [53, 54]. Studies in INS-1 cells have also indicated that betacellulin- (BTC-) mediated transactivation of the epidermal growth factor receptor/erb-B1 is a prerequisite for GLP-1-induced proliferative effects in these cells [49]. However, BTC failed to induce proliferation in a different insulinoma cell line, RINm5F and also in fetal human beta cells [55]. The importance of the Pdx1 transcription factor in mediating the proliferative effects of GLP-1 in beta cells was demonstrated using mice with beta cell-specific inactivation of the Pdx1 gene. Ex-4-mediated proliferation was blocked in isolated islets from these mice, suggesting that Pdx1expression is essential for Ex-4-induced proliferative effects [51]. Interestingly, Ex-4 induces Pdx1 expression in human fetal islet cell cultures and promotes functional maturation and proliferation of human islet cell cultures transplanted under the rat kidney capsule [56]. It has become clear that GLP-1 acts by means of Gs*α* and PI-3K/PKB to stimulate beta cell proliferation and survival. A beta-cell-specific Gs*α* deficiency in mice results in diabetes characterized by reduced insulin secretion and beta-cell mass with the primary defect being in decreased beta-cell proliferative capacity [57]. It has been also shown that GLP-1 inhibits FoxO1 transcriptional activity through phosphorylation-dependent nuclear exclusion in beta cells [15], and the ability of Ex-4 to increase beta-cell mass was blunted in transgenic mice expressing constitutively nuclear FoxO1 in beta cells [15]. FoxO1 inactivation plays an important role in the effect of GLP-1 on the expression of the two important transcription factors PDX-1 and Foxa2 [16]. GLP-1 activation of PI-3K/PKB facilitates acute nuclear translocation of existing PDX-1. Indeed, mice with a beta-cell-specific inactivation of PDX-1 do not display a proliferative response to Ex-4 treatment [51]. GLP-1 activation of PI-3K is mediated by transactivation of EGFRs via GLP-1R-mediated activation of c-Src that in turn activates a membrane-bound metalloproteinase, with concomitant release of the soluble ligand BTC which is an agonist of EGFRs [49]. This is also followed by activation and translocation to the nucleus of PKC*ζ*, resulting in enhancement of the stimulatory effect of GLP-1 on beta-cell proliferation [53]. GLP-1 also exerts its stimulatory effects on beta-cell proliferation through CREB-mediated Irs2 gene expression, leading to activation of PI-3K/PKB [58]. GLP-1R activation has been shown to upregulate the expression of cyclin D1 [59, 60], and this effect is likely to be mediated by PKA-dependent activation of CREB [60, 61]. A recent study showed that GLP-1R signaling via cAMP/PKA activates *β*-catenin/T-cell factor-like 2- (TCF7L2-) dependent Wnt signaling in the proliferation through upregulation of cyclin D1 [62]. A prominent role for *β*-cateninTCF7L2-dependent Wnt signaling is now acknowledged after the reports that GSK-3*β* overexpression in mice induces beta cell mass restriction and the development of diabetes [63], that genetic disruption of GSK-3*β* in beta-cells results in increased beta-cell mass and that beta-cell regeneration can be promoted by systemic administration of GSK-3*β* inhibitors to streptozotocin-induced neonatal diabetic rats [64]. Unexpectedly, the proliferative effect of GLP-1 was recently related to IGF-1R expression and autocrine secretion of IGF-2 by the beta-cell, since this effect was suppressed by Igf-1r gene inactivation and by IGF-2 immunoneutralization or knockdown [43]. 

GLP-1R activation reduces beta-cell apoptosis in purified rodent and human islets as well as beta-cell lines after exposure to many cytotoxic agents, including reactive oxygen species, glucose, free fatty acid, palmitate, cytokines, tumor necrosis factor-*α* (TNF-*α*), immunosuppressive reagents, and dexamethasone [66]. A role for endogenous GLP-1 in prevention of beta-cell death was demonstrated by the increased susceptibility to streptozotocin-induced apoptosis in GLP-1R knockout mice; conversely, streptozotocin-induced apoptosis was significantly reduced by coadministration of Ex-4 [67]. Ex-4 also reduces biochemical markers of islet ER stress in vivo and ER stress-associated beta-cell death in a PKA-dependent manner [68, 69]. Similar to GLP-1-induced beta-cell proliferation, antiapoptotic effects of GLP-1 in beta cells are mediated by promotion of FoxO1 nuclear exclusion and consequent upregulation of PDX-1 and Foxa2 expression via EGFR- and PI3K-dependent activation of PKB and cAMP/PKA-dependent activation of CREB, leading to upregulation of IRS2 protein expression and activation of PKB. Also similar to the induction of proliferation, activation of IGF-1 receptor expression and IGF-2 secretion participate to the GLP-1-induced the protection of the beta cells against cytokine-induced apoptosis, through [43]. The protective effect of GLP-1 on beta-cell glucolipotoxicity is also mediated by PKB activation and possibly its downstream target nuclear factor-*κ*B [70]. In addition, recent studies suggest that GLP-1R agonists protect beta cells from proinflammatory cytokine-induced apoptosis by inhibiting the c-Jun NH2-terminal kinase pathway via upregulation of islet-brain 1, a potent blocker of the c-Jun NH2-terminal kinase pathway [71], and activation of the extracellular signal-regulated kinase 1/2-dependent pathway [72]. 

GLP-1R was confirmed to be present in pancreatic ducts in mouse, rat, and human [73, 74]. GLP-1R exists in the AR42J acinar cell line derived from a rat pancreatic tumor and treatment with GLP-1 or Ex-4 causes increases in both intracellular cAMP and Ca^2+^ levels [75]. Activation of GLP-1R signaling either in ductal or acinar cell lines or in vivo in rodents has resulted in differentiation of a fraction of these cells toward an islet-like phenotype, in association with activation of PKC and MAPK, and transcription factors necessary for an endocrine phenotype such as PDX-1, as well as the glucose-sensing factors glucokinase and GLUT2 [76]. GLP-1R activation of those cells also affects transforming growth factor-*β* signaling pathways, resulting in reduced Smad activity [77, 78]. AR42J cells, even without GLP-1R activation, have the potential to be converted into endocrine [79] but are negative for islet hormones and their transcripts under usual culture conditions [76]. When these cells were exposed to GLP-1 or Ex-4, approximately 20% of the cells contained insulin protein and were capable of releasing insulin in a glucose-mediated mode [76]. Such GLP-1 effect was also observed in Capan-1 cell line [80] and rat ARIP and human PANC-1 cell lines. Similar to the AR42J cells, GLUT2 and glucokinase transcripts were induced in these cell lines [81]. In particular, the differentiation-promoting activity of GLP-1 requires the expression of PDX-1, because PANC-1 cells, which lack endogenous PDX-1, differentiate only when transfected with PDX-1, whereas rat ARIP cells that express PDX-1 are susceptible to undergoing differentiation into insulin-secreting cells [81]. In the Capan-1 cell line, differentiation to insulin-producing cells was also seen when they were transfected with PDX-1, and PDX-1 antisense totally inhibited such conversion [80]. In human pancreatic ducts also where GLP-1 receptor is abundantly expressed, Ex-4 treatment in vitro increases the number of insulin-producing cells. This suggests that GLP-1/Ex-4 is useful to facilitate beta-cell neogenesis in adult pancreatic ducts [74].

## 5. Pharmacological Activation of the GLP-1R Signalling Pathway in Models of Deficient Beta-Cell Mass

**Figure 1:**
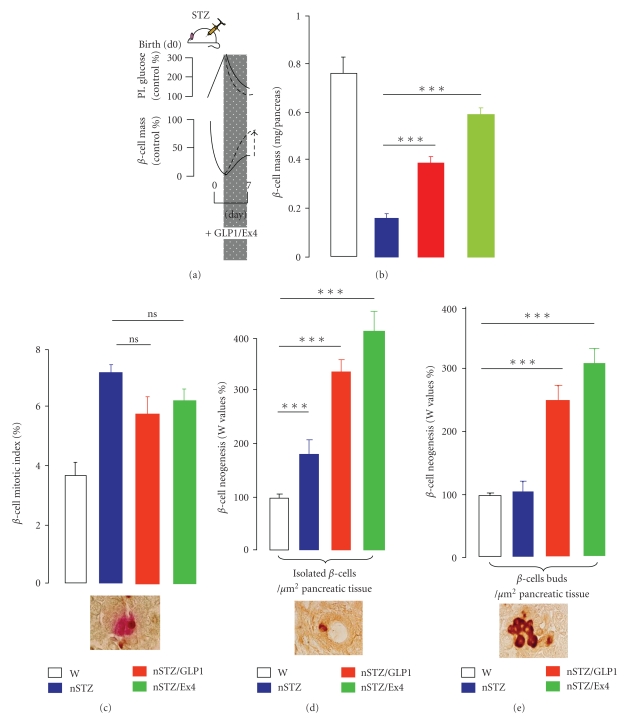
GLP-1 or Exendin-4 (Ex-4) activates beta-cells regeneration in vivo. The aim was to investigate in the rat model of neonatal beta-cell regeneration (nSTZ model), the capacity of in vivo treatments with GLP-1 or Ex-4 to promote beta-cell regeneration. To this end, nSTZ rats from the Wistar strain (W) were submitted to GLP-1 or Ex-4 administration from postnatal day 2 to day 6 only, and their beta-cell masses were tested on day 7 (a) and (b). In the nSTZ/GLP-1 and nSTZ/Ex-4 groups, total beta-cell masses per pancreas were both significantly increased (***P < .001) as compared with values in untreated nSTZ rats, representing, respectively, 51% and 71% of the control Wistar beta-cell mass, while nSTZ beta-cell mass represented only 21% of the control Wistar value. Beta-cell BrdU labeling index (c) in the untreated nSTZ rats was found to be significantly increased (*P* < .001) as compared with Wistar group. In the nSTZ/GLP-1 and nSTZ/Ex-4 groups, it was similarly increased. A representative figure is given with double immunostaining for BrdU and insulin in 7-day-old nSTZ rats (magnification ×1000). To estimate activation of neogenesis (d) and (e), the number of single beta-cells incorporated into the duct epithelium and the number of beta-cell clusters budding from ducts were quantified. The number of isolated beta-cell within pancreatic tissue of nSTZ rats represented 185% of Wistar value and the number of beta-cell buds in pancreatic tissue in nSTZ rats represented 106% of Wistar value. These two parameters were strongly increased in nSTZ/GLP-1 and nSTZ/Ex-4 rats as compared to untreated nSTZ rats (***P < .001). A representative figure is given with indirect immunoperoxydase staining for insulin in 7 day-old nSTZ/GLP-1 rats (magnification ×1000). Adapted from Tourrel et al. [83].

**Figure 2:**
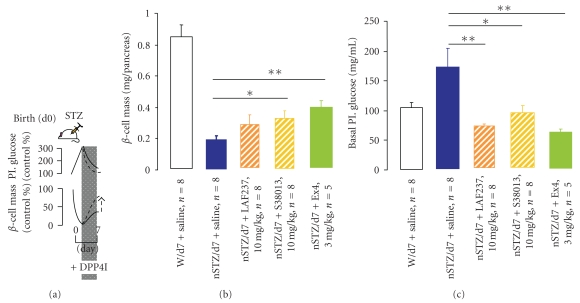
DPPIV inhibitors activate beta-cells regeneration in vivo. The aim was to investigate in the rat model of neonatal beta-cell regeneration (nSTZ model), the capacity of in vivo treatment by DPPIV inhibitors (LAF237 or S38013) to promote beta-cell regeneration. To this end, nSTZ rats were submitted to DPPIV administration from postnatal day 2 to day 6 only, and their beta-cell masses were tested on day 7. Ex-4 was taken as a beta-cell growth stimulator comparator. In the 7-day-old untreated nSTZ group, total beta-cell mass per pancreas was only 22% of the value in the untreated normal group (*P* < .001). In the nSTZ/LAF237 group, the total beta-cell mass increase (by 46%) did not reach statistical significance. In the nSTZ/S38013 group, the total beta-cell mass increase (by 68%) reached statistical significance (*P < .05). Beta-cell mass in the nSTZ/Ex-4 group was twice increased (**P < .01). In the 7-day-old untreated nSTZ group, basal plasma glucose value was significantly increased by 70% (*P* < .05) as compared to that of untreated normal group. By contrast, in the nSTZ/LAF237, nSTZ/S38013, and nSTZ/Ex-4 groups, basal plasma glucose levels were significantly decreased (*P* < .05 or *P* < .01) at the end of treatment as compared with those in the untreated nSTZ group and reached values no longer significantly different from those in untreated normal pups.

Acute or chronic treatment of diabetic rodents with GLP-1R agonists stimulates beta-cell proliferation and neogenesis and slows the rate of beta-cell apoptosis, leading to an expansion of beta-cell mass. In vivo administration of GLP-1, Ex-4 or other degradation-resistant analogs has been shown to increase beta-cell mass in different prediabetic and diabetic rodent models [11, 65, 82–86]. Administration of GLP-1 or Ex-4 for several days resulted in stimulated expansion of beta-cell mass and increased beta-cell proliferation in old glucose-intolerant rats [84], adult db/db mice, Zucker rats, pancreatectomized rats and mice, or intrauterine growth-retarded rats [87]. A transient treatment of GLP-1 or Ex-4 in STZ-treated newborn rats resulted in a sustained improvement of beta-cell mass through increased beta-cell neogenesis and replication [83] (Figure 1). We obtained similar conclusion in the nSTZ model after in vivo administration of a DPPIV inhibitor (Figure 2). Moreover, diabetic Lepdb/Lepdb mice treated with Ex-4 for 2 weeks showed enhanced expression of PDX-1 in the ducts (favoring the presence of GLP-1R in ductal cells, as referenced above) and the exocrine tissue [11], which means that GLP-1R agonists aid in islet neogenesis, because ductal cells have been thought to be the main source for endocrine neogenesis [88].

Taking advantage of the GK/Par rat model of spontaneous T2D, we have raised the question of what is the impact of GLP-1 or Ex-4 treatment, in terms of beta-cell mass enlargement and long-term improvement of glucose homeostasis. To address this issue, we investigated the ability of GLP-1 or Ex-4 treatment to promote beta-cell proliferation in young GK/Par rats during the prediabetic stage and thereby to prevent the pathological progression of the T2D when animals become adults. GK/Par rats were submitted to GLP-1 or Ex-4 injection from postnatal day 2 to day 6 only [84]. Both treatments enhanced, on day 7, pancreatic insulin contents and total beta-cell mass by stimulating both beta-cell neogenesis and beta-cell regeneration. Followup of biological characteristics from day 7 to adult age (2 months) showed that both treatment exerted long-term favorable influence on beta-cell mass and glycaemic control at adult age. As compared to untreated GK/Par rats, 2-month-old GLP-1 or Ex-4-treated GK rats exhibited improved glucose-stimulated insulin secretion, in vivo after intravenous glucose load or in vitro using isolated perfused pancreas. Moreover, plasma glucose disappearance rate was increased in both treated GK/Par groups compared to untreated GK/Par group [84]. These findings model indicate that a GLP-1 or Ex-4 treatment limited to the prediabetic period, delays the installation, and limits the severity of T2D in the GK/Par model.

GLP-1R activation also promotes preservation and expansion of beta-cell mass in type 2 diabetic rodent models through protecting beta cells against the deleterious effects of the diabetic milieu (i.e., increased cytokine toxicity, glucose toxicity, and lipotoxicity). Ex-4 treatment of Lepdb/Lepdb mice decreases activation of caspase-3 and prevents beta-cell apoptosis through PKB and MAPK [85] and infusion with GLP-1 drastically reduced the number of apoptotic beta cells in islets of Zucker diabetic rats [65]. 

Collectively, the studies above mentioned so far indicate that GLP-1R agonists may prove useful for expansion of human beta cells either cultured in vitro, after transplantation, or after sustained treatment of diabetic subjects in vivo.

## 6. Current Issues That Challenge the Beneficial Effects of GLP-1R Agonists for Beta-Cell Therapy

### 6.1. Risk of Pancreatic Tumor Formation in Patients Receiving GLP-1R Agonist Supplementation

If GLP-1R agonists were to be used continuously to treat diabetes, then uncontrolled beta-cell proliferation would become an issue unless there were brakes on the system. Klinger et al. [89] shed light on cellular mechanisms that may indeed limit the proliferative effect of GLP-1 in beta-cells: GLP-1 provides its own brakes because it leads to the rapid and strong expression of four negative regulators of intracellular signalling: RGS2 (regulator of G protein signalling 2), Dusp14 (dual-specificity phosphatase 14, also called MAP kinase phosphatase 6, a negative feedback regulator of the mitogen-activated protein kinase signaling cascade), Icer (inducible cAMP early repressor), and Crem-*α* (cAMP responsive element modulator alpha). However, an obvious question to be further studied is whether unrestrained beta-cell proliferation may result from loosening of the GLP-1 effect upon these negative regulators of beta-cell growth, during long-term treatment.

### 6.2. Risk of Pancreatitis in Patients Receiving GLP-1R Agonist Supplementation

Another concern with respect to clinical use of GLP-1 relates to reports of pancreatitis in some patients long-term treated with long-acting GLP-1 receptor agonists [90]. However, amylase levels and pancreatic markers of inflammation were found reduced in Ex-4-treated mice, and Ex-4 did not increase the severity of pancreatitis in a murine model of this condition [91]. Furthermore, no evidence was found for an increased incidence of pancreatitis in a large cohort of patients treated with either exenatide or sitagliptin, compared with those treated with metformin or glyburide [92]. Notwithstanding, a recent paper in the HIP diabetic rat model treated with sitagliptin has reported ductal cell hyperplasia in all sitagliptin-treated animals, acinar to ductal metaplasia in some, and haemorrhagic pancreatitis in one isolated case [93]. Histologic evidence of pancreatic acinar inflammation has also been found in rats treated with Ex-4 and their acinar cells were abnormal in appearance and had a greater frequency of cell death [94]. Pancreatic ductal replication is increased in humans with obesity and/or type 2 diabetes [95], providing a possible link between the increased risk of pancreatitis in individuals with obesity and/or T2D. In common with the HIP rat model of diabetes, pancreatic ductal replication was also increased in humans with obesity and T2D [95]. The mechanisms that induce increased pancreatic ductal replication in patients with obesity and/or type 2 diabetes are unknown. Excessive fat accumulation in pancreas could induce local inflammation [95]. Increased beta cell apoptosis in type 2 diabetes is also associated with inflammation and increased local cytokines [96]. Such combination might activate islet regeneration via duct-related progenitors, comparable to the process proposed for acinar tissue in chronic pancreatitis. GLP-1 therapy may potentially amplify ductal hyperplasia since it has been reported to activate pancreatic regenerative efforts with increased duct cells positive for PDX-1 [74]. Given the clinical gravity of pancreatitis, a better understanding of this issue is important.

### 6.3. Relevance of GLP-1R Agonist Supplementation for T1D

Although regenerative and antiapoptotic actions of GLP-1 or Ex-4 have been demonstrated in both normoglycemic and diabetic animal models, the majority of these studies were conducted in animal models of T2D [83–87]. In contrast, much less is known about whether the actions of GLP-1R agonists are maintained in the setting of an ongoing autoimmune attack, as is the case in the NOD mouse, the BB rat, and in human subjects with type 1 diabetes (T1D). Zhang et al. [97] have shown that continuous delivery of GLP-1 via an osmotic minipump in prediabetic NOD mice results in significant increases in beta-cell mass and replication rate, together with a significant reduction in the rate of beta-cell apoptosis. Hence, it seems possible that GLP-1R activation may be able to enhance beta-cell mass even in the presence of an autoimmune attack, if therapy is initiated before the onset of hyperglycemia. Ex-4 has also been administered to NOD mice alone or in combination with different immune modulators, lisofylline [98], antilymphocyte serum [99], or anti-CD3 immunotherapy [100]: the highest frequency of diabetes remission was observed in animals that received the combination treatments, suggesting a beneficial synergistic effect between immunomodulators and the Ex-4 regenerative agent. Furthermore, recent studies have reported that increasing the levels of circulating GLP-1 by inhibiting dipeptidyl peptidase-4 results in prolonged islet graft survival and decreased insulitis in diabetic NOD mice [101], that Ex-4 in vitro decreased IFN-*γ*-induced expression of several inflammatory mediators in human islets and MIN6 cells [102] and that Ex-4 in vivo induced a recovery of beta-cell proliferation during the initial stages of insulitis in the BB/Worcester rat [103]. The finding of an anti-inflammatory action of Ex-4 may have implications for the treatment of both types of diabetes, since the presence of immune cells in islets from human T2D diabetic patients and from animal models of T2D has been reported [96].

### 6.4. No Reliable Method to Assess Beta-Cell Mass in Patients Receiving GLP-1R Agonist Supplementation

Among the demonstrated biological actions of GLP-1R agonists, none has generated more interest than the findings of enhanced beta-cell growth and survival in rodent diabetic models [104]. The possibility that incretin therapy may not only improve beta-cell function but also increase beta-cell mass in patients with T2D has, therefore, created much excitement. Determination of the success of therapeutic strategies designed to enhance beta-cell regeneration requires reliable methods for the assessment of beta-cell mass. In animal models, beta-cell mass can be easily calculated as the product of pancreatic weight and the fractional beta-cell area in cross-sections from different regions of the pancreas. In human, there are no direct measures currently available to determine whether an antidiabetes drug has the ability to alter the course of T2D by increasing beta-cell mass (replication, neogenesis) or attenuating beta-cell apoptosis. Noninvasive imaging techniques that can assess islet mass are currently being explored, but have not yet reached the sensitivity that is required for use in humans, and pancreatic tissue for histological examination cannot be ethically procured for research purposes only. This leaves no direct means for testing direct effects of GLP-1-based drugs on beta-cell mass in diabetic patients.

### 6.5. Significance of the Islet-Derived GLP-1 Source and Its Modulation by GLP-1R Agonist Supplementation

Beside production and secretion of GLP-1 by the enteroendocrine L cells throughout the intestinal epithelium, there is now growing evidence that under certain conditions, islet alpha-cells are an extraintestinal site for GLP-1 production, perhaps to support the function and/or survival of neighboring beta-cells. While proglucagon is expressed in islet alpha-cells, PC2 is the predominant processing enzyme in these cells, cleaving proglucagon to yield glucagon rather than GLP-1. However, under certain conditions, alpha cells do express PC1/3 and liberate GLP-1 from proglucagon instead. Several models of pancreatic injury have been associated with islet GLP-1 production. Treatment of neonatal rats with STZ increases pancreatic GLP-1 content [105]. STZ treatment of adult rats increases PC1/3 expression in glucagon-immunoreactive cells in islets and increases GLP-1 levels in islets and plasma [106]. It has been shown recently that treatment of isolated mouse islets with a PC1/3-expressing adenovirus induces GLP-1 release from alpha cells, increases GIIS, and promotes islet survival [107]. In addition, transplantation of PC1/3-expressing alpha-cells increases plasma GLP-1 levels and improves glucose homeostasis in rodent models of type 1 and type 2 diabetes [108]. Thus, manipulation of proglucagon processing in the alpha cell to yield GLP-1 can be wiewed as a startegy for enhancing islet function and survival. Since GLP-1 seem to be expressed in islets under certain conditions, it might be necessary to revise our understanding of how this hormone modulates beta-cell secretion and growth. Our current view relies on the network of neural and endocrine signals originating in the gut after food intake that stimulate insulin secretion. We should now also consider the possibility that intraislet GLP-1 signals might modulate insulin secretion and/or influence beta-cell survival. Determining the physiological importance of the islet-derived GLP-1 source during diabetes and its modulation during administration of exogenous GLP-1R agonists is a clinically relevant issue.
